# Blood Pressure-Lowering Effect of Wine Lees Phenolic Compounds Is Mediated by Endothelial-Derived Factors: Role of Sirtuin 1

**DOI:** 10.3390/antiox10071073

**Published:** 2021-07-03

**Authors:** Raúl López-Fernández-Sobrino, Jorge R. Soliz-Rueda, Javier Ávila-Román, Anna Arola-Arnal, Manuel Suárez, Begoña Muguerza, Francisca Isabel Bravo

**Affiliations:** Nutrigenomics Research Group, Department of Biochemistry and Biotechnology, Universitat Rovira i Virgili, 43007 Tarragona, Spain; raul.lopez@urv.cat (R.L.-F.-S.); jorgericardo.soliz@urv.cat (J.R.S.-R.); franciscojavier.avila@urv.cat (J.Á.-R.); anna.arola@urv.cat (A.A.-A.); manuel.suarez@urv.cat (M.S.); franciscaisabel.bravo@urv.cat (F.I.B.)

**Keywords:** endothelial function, endothelial nitric oxide synthase, endothelin-1, NADPH oxidase, spontaneously hypertensive rats

## Abstract

The antihypertensive effect of wine lees powder (WLPW) from a Cabernet grape variety was related to its high content in flavanols and anthocyanins compounds. This study investigates the involvement of endothelial-derived factors and SIRT1 in its bioactivity. Spontaneously hypertensive rats (SHR) were orally administered water or WLPW (125 mg/kg bw). Posteriorly, both groups were intraperitoneally administered saline, Nω-nitro-L-arginine methyl ester (L-NAME), a nitric oxide (NO) synthesis inhibitor, indomethacin, a prostacyclin synthesis inhibitor, or sirtinol, an inhibitor of sirtuins. Blood pressure (BP) was recorded before and 6 h after WLPW administration. In an additional experiment, SHR were administered water or WLPW and endothelial expressions of *eNos*, *Sirt1*, *Nox4*, and *Et1* were determined. The BP-lowering properties of WLPW were abolished by L-NAME and partially reduced by indomethacin, demonstrating that WLPW antihypertensive effect was mediated by changes in NO availability, although prostacyclin also contributed to this activity. Moreover, BP-lowering effect was reduced by sirtinol, indicating that WLPW decreased BP in a SIRT1-dependent manner. Furthermore, WLPW upregulated *eNos* and *Sirt1* and downregulated *Nox4* and *Et1* endothelial gene expression. These results evidence the vasoprotective effect of WLPW and show that its antihypertensive effect in SHR is endothelium dependent and mediated by SIRT1.

## 1. Introduction

Endothelium plays an important role in the regulation of vascular tone and blood fluidity by balancing the production of endothelium-derived vasodilator and vasoconstrictor factors [1]. Alterations in its functionality, called endothelial dysfunction, are associated to different diseases including hypertension (HTN). This dysfunction produces an imbalance between vasodilator and vasoconstrictor factors by decreasing the availability of vasodilators, mainly nitric oxide (NO) and/or increasing the production of vasoconstrictor factors [2].

NO is the main endothelial-derived vasodilator factor involved in vascular tone regulation [3]. Its endothelium production is mediated by the endothelial NO synthase (eNOS), that converts L-arginine in NO [4]. Activation of this enzyme depends on intracellular Ca^2+^ levels, which mediate eNOS detachment from caveolin [5]. In response to oxidative stress, endothelium-derived NO production is enhanced by Sirtuin 1 (SIRT1), a NAD^+^-dependent deacetylase, which increases *eNos* transcription and enzymatic activity of eNOS via its deacetylation [6,7]. Furthermore, SIRT1 also decreases the NADPH oxidase (NOX)-dependent production of reactive oxygen species (ROS), acting on gene expression and activity of the vascular NOX subunits p22phox and 4 (NOX4) [8]. An excess in ROS levels leads to endothelial dysfunction and vasoconstriction since ROS react to NO, producing peroxynitrite and decreasing NO bioavailability [2]. In addition to NO, prostaglandin I2 (PGI2), namely prostacyclin, is also an important vasodilator implicated in endothelial regulation [9]. However, it seems that PGI2 exerts its effect when NO levels are reduced [10]. Endothelial PGI2 is produced by cyclooxygenase (COX) isomer 2, which catalyzes the conversion of arachidonic acid to prostaglandin H2 (PGH2). PGH2 is further transformed into PGI2 by prostacyclin synthase [11]. In contrast to endothelial-derived relaxing factors, endothelin 1 (ET-1) is an endothelial-derived vasoconstrictor factor, which is overexpressed in the vasculature of different hypertensive models [12]. ET-1 is produced by the proteolytic cleavage of its precursor, big ET-1, by endothelin converting enzyme [13].

We have previously demonstrated the antihypertensive effect of wine lees (WL) from Cabernet grape variety in spontaneously hypertensive rats (SHR). The BP-lowering effect was related to the higher content of flavanols and anthocyanins present in these WL with respect to the other varieties studied [14]. A dose of 125 mg/kg body weight (bw) of dealcoholized WL powder (WLPW), which would be equivalent to 1.8 g/day in humans, caused an antihypertensive effect more potent than that obtained with the antihypertensive drug captopril [15]. This potent BP-lowering property was associated to an improvement of oxidative stress state of WLPW-treated animals and attributed to its high content in flavanols and anthocyanins [15]. Nevertheless, different studies have shown that grape phenolic compounds can also exert their antihypertensive effect through an improvement of endothelial dysfunction. In this regard, Kondrashov et al. observed an increase in eNOS activity after an acute administration of a red wine extract to SHR [16]. In addition, the long-term administration of a grape seed proanthocyanidin extract (GSPE) to diet-induced hypertensive rats produced an antihypertensive effect and conferred a vasoprotective pattern, including an overexpression of endothelial *Sirt1* [17]. According to this, the acute antihypertensive effect of GSPE in SHR was completely abolished by sirtinol, an inhibitor of sirtuins, indicating that grape seed flavanols decrease BP in a SIRT1-dependent manner. Thus, the aim of this study was to evaluate the involvement of endothelial-derived factors on the BP-lowering effect of WLPW and to study a potential role of SIRT1.

## 2. Materials and Methods

### 2.1. Obtaining and Characterization of Wine Lees

WL from grapes of Cabernet variety were provided by Grandes Vinos y Viñedos, S.A, located in the Cariñena P.O.D area (Zaragoza, Spain). WL were centrifuged at 3000× *g* for 15 min at 4 °C. The supernatant was collected, freeze-dried, and ground to obtain the WLPW. Finally, WLPW was kept at room temperature and protected from light exposure and humidity until its administration to animals.

Humidity, total protein content, measured by Kjeldahl method, total phenolic content, measured by Folin-Ciocalteu method, and the antioxidant capacity, measured by DPPH method, of WLPW were 7.85 ± 1.49%, 222.61 ± 5.73 mg/g of wet weight, 76.40 ± 0.74 mg gallic acid equivalents (GAE)/g of wet weight and 5.50 µg/mL as IC_50_ of antioxidant capacity, respectively [15]. Supplementary Tables S1 and S2 show the WLPW phenolic profile. Individual phenolic compounds were characterized by using a high-performance liquid chromatography coupled to electrospray ionisation and quadrupole time-of-flight mass spectrometry (UHPLC-ESI-Q-TOF-MS) system using 1290 UHPLC Infinity II series coupled to a Q-TOF/MS 6550 (Agilent Technologies, Palo Alto, CA, USA). Both negative and positive ionization ([M−H]^−^ or [M−H]^+^) were used to identify parental ions and fragmentation patterns.

### 2.2. Dosage Regimen and Experimental Procedure in Animal

**Figure 1:**
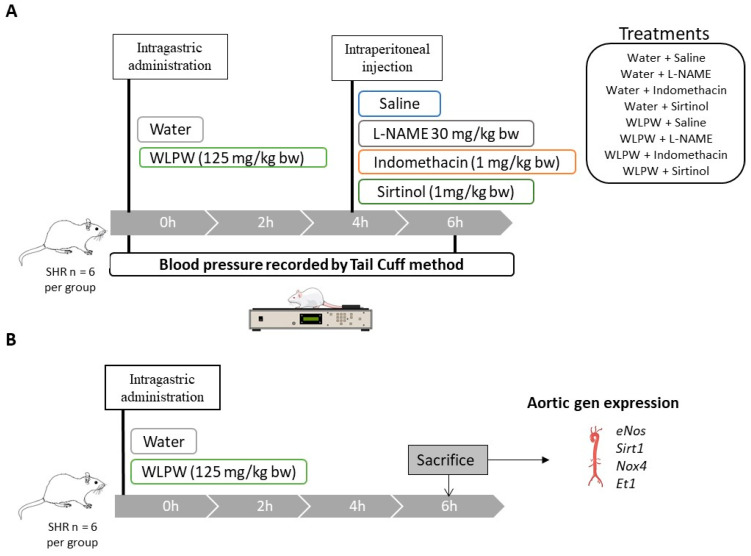
Graphical representation of the experimental design for Nω-nitro-L-arginine methyl ester hydrochloride (L-NAME), indomethacin and sirtinol study in spontaneously hypertensive rats (SHR) (**A**), and graphical representation of the experimental design used to study the effects of wine lees powder (WLPW) on the endothelial function (**B**).

Male SHR (19–22 weeks old) weighing between 340–390 g and purchased from Charles River Laboratories España S.A. (Barcelona, Spain) were used in this study. They were singly housed in animal quarters at 22  °C and 50% of humidity with a light/dark period of 12  h. Tap water and standard diet (A04 Panlab, Barcelona, Spain) were provided ad libitum during the experiments. Figure 1 shows a graphical representation of the two experimental designs used in this study.

Rats were administered tap water or a single dose of 125 mg/kg bw WLPW (human equivalent dose 1.8 g/day) dissolved in tap water by oral gavage between 8:00 and 9:00 a.m. Total volume administered to rats was 1.5 mL. Four hours after oral administration of these treatments, animals were intraperitoneally administered with 1 mL of saline solution, 30 mg/kg bw L-NAME (PubChem CID: 135193; Sigma-Aldrich, Barcelona, Spain), 5 mg/kg bw indomethacin (PubChem CID: 3715; Sigma-Aldrich, Barcelona, Spain), or 1 mg/kg bw sirtinol (PubChem CID: 2827646; Sigma-Aldrich, Barcelona, Spain), obtaining the following working groups: Water + Saline, Water + L-NAME, Water + Indomethacin, Water + Sirtinol, WLPW + Saline, WLPW + L-NAME, WLPW + Indomethacin, WLPW + Sirtinol (*n* = 6 per group). All treatments were prepared in saline solution.

Systolic and diastolic BP (SBP and DBP respectively) were measured by tail cuff method following the method described by Quiñones et al. [18] before and 6 h after oral administration of water or WLPW to SHR. Before the BP measurements, animals were maintained at 38 °C for 15 min to detect the pulsations of their tail arteries. To guarantee the reliability of the results, five continuous measurements were used to determine the SBP and DBP of animals. In addition, to minimize stress-induced variations in BP, animals were trained for 2 weeks prior to the procedure after a 10-day adaptation period and all measurements were taken in a peaceful environment and by the same person.

In the second study, male SHR (19–22 weeks old) weighing 336–392 g and purchased from Charles River Laboratories España S.A. (Barcelona, Spain) were also used. Diet and housing conditions were the same as the first mentioned study. Animals were divided in two groups and were administered tap water (1.5 mL) or WLPW (125 mg/kg bw, dissolved in 1.5 mL of tap water) by oral gavage (*n* = 6 per group). Six hours post-administration, animals were sacrificed by live decapitation. Aorta was excised and immediately frozen in liquid nitrogen for further study.

The animal protocols followed in this study were conducted in accordance with the European Communities Council Directive (86/609/EEC) and approved by the Animal Ethics Review Committee for Animal Experimentation of the Universitat Rovira i Virgili. Moreover, it was further approved by Comissió d’Experimentació Animal, body authorized by the Generalitat de Catalunya (Spain), with the identification code of 10,780 on 26/05/2020. This protocol was linked to the project Retos Colaboración: RTC-2017-6044-2 from the Spanish Ministry of Economy and Competitiveness and European Regional Development Fund (FEDER).

### 2.3. RNA Extraction and mRNA Quantification by Real-Time qPCR

The frozen aorta was homogenized in a TissueLyser (Qiagen, Barcelona, Spain) without buffer and with stainless steel balls. Then, lysis buffer was immediately added for RNA extraction using the RNeasy Mini Kit (RNeasy Mini Kit, Qiagen). Total extracted RNA was quantified using a Nanodrop 100 Spectrophotometer (ThermoFisher Scientific, Madrid, Spain).

mRNA reverse transcription was carried out by using the High Capacity cDNA Reverse Transcription Kit (AppliedBiosystems, Madrid, Spain). Quantitative PCR amplification and detection were performed in a 96-well plate using SYBR PCR Premix Reagent Ex Taq™ (Takara, Barcelona, Spain) and the CFX96 Touch Real Time PCR System (Bio-Rad, Barcelona, Spain) following the manufacturer procedures.

Relative mRNA levels of *eNos*, *Sirt1*, *Nox4*, and *Et1* were analyzed by real-time PCR using peptidyl prolyl isomerase A (*Ppia*) as the housekeeping gene. Table 1 shows the primers used, which were obtained from Biomers (Söflinger, Germany). Melting curve analysis and gel electrophoresis separation (3% agarose) were used to verify primer specificity and amplicon size, respectively. qPCR efficiency was calculated by evaluating a 2-fold dilution series of aortic cDNA and calculated by E = 10(1/slope). The results were expressed as the logarithm of the cDNA concentration vs. the obtained Ct. The relative expression or relative quantification was calculated by RQ = (E_target_)^∆Ct(target)^/(E_reference_)^∆Ct(reference)^ where ∆Ct (target) = Ct (target gene calibrator) − Ct (target gene in test) and ∆Ct (reference) = Ct (reference gene calibrator) − Ct (reference gene in test). Each sample was performed at least in duplicate.

### 2.4. Statistical Analysis

BP differences produced by the treatments were analysed by a one-way analysis of variance (ANOVA). Student’s *t*-test was used to evaluate differences between groups in gene expression. All the analyses were performed using GraphPad Prism 7.04 for Windows (GraphPad Software, San Diego, CA, USA). Outliers were determined by using Grubbs’ test. Differences between groups were considered significant when *p* < 0.05.

## 3. Results

### 3.1. Role of Nitric Oxide, Prostacyclin and Sirtuin in the BP-Lowering Effect of WLPW

**Figure 2:**
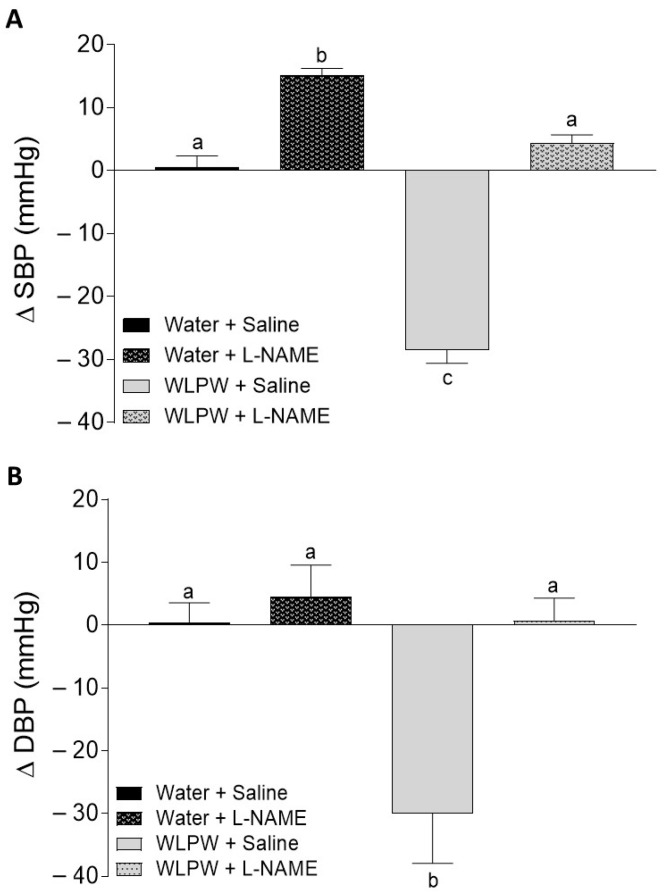
Changes in systolic blood pressure (SBP) (**A**) and diastolic blood pressure (DBP) (**B**) caused in spontaneously hypertensive rats 6 h post-administration by different treatments: oral administration of water or wine lees powder (WLPW) and intraperitoneal injection of Saline or Nω-nitro-L-arginine methyl ester hydrochloride (L-NAME). Significant differences (*p* < 0.05) are represented by different letters and *p* was estimated by one-way ANOVA.

**Figure 3:**
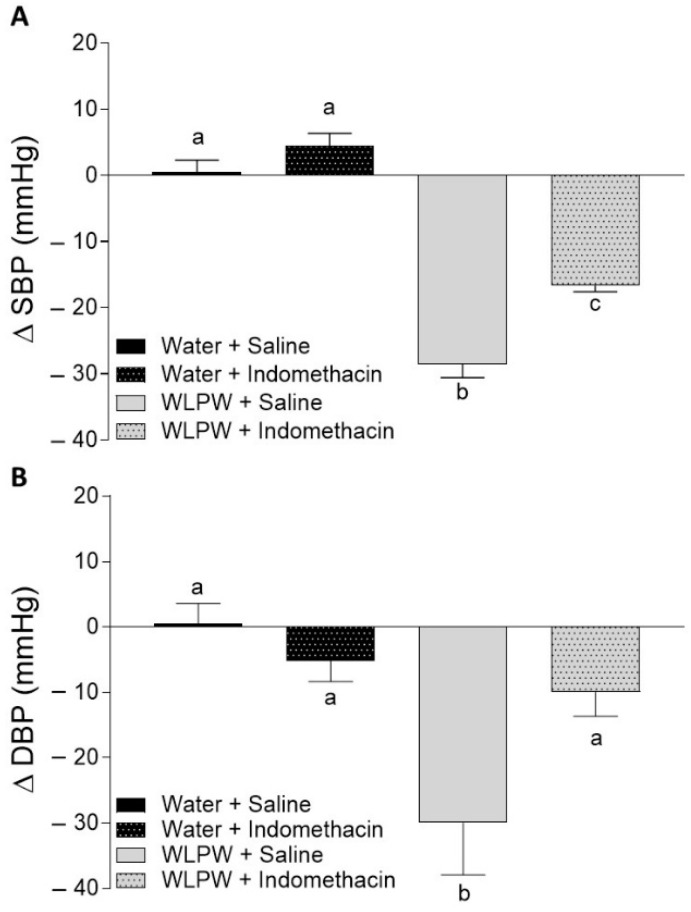
Changes in systolic blood pressure (SBP) (**A**) and diastolic blood pressure (DBP) (**B**) caused in spontaneously hypertensive rats 6 h post-administration by different treatments: oral administration of water or wine lees powder (WLPW) and intraperitoneal injection of Saline or Indomethacin. Significant differences (*p* < 0.05) are represented by different letters and *p* was estimated by one-way ANOVA.

**Figure 4:**
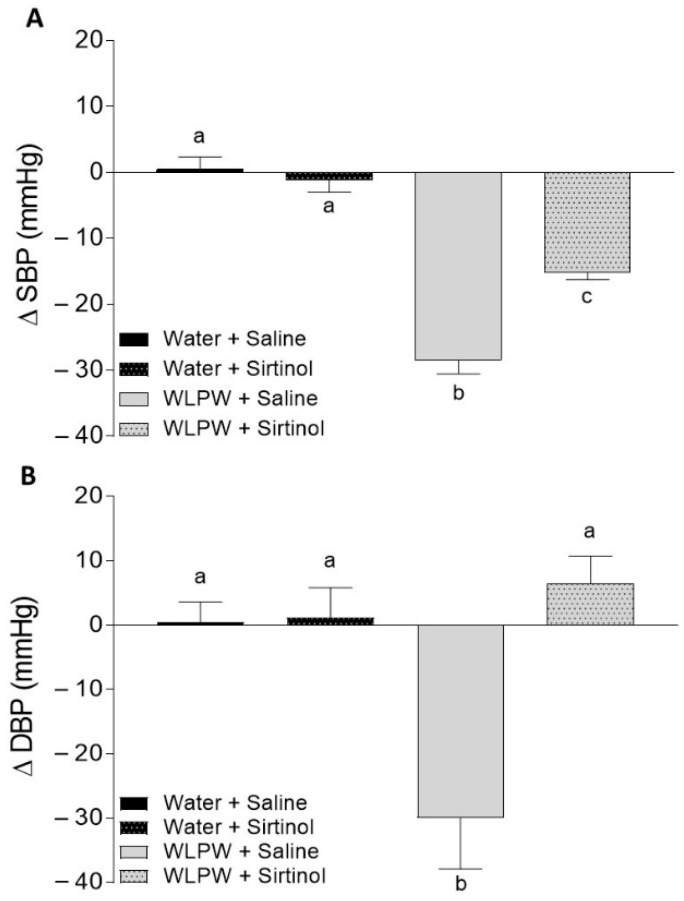
Changes in systolic blood pressure (SBP) (**A**) and diastolic blood pressure (DBP) (**B**) caused in spontaneously hypertensive rats 6 h post-administration by different treatments: oral administration of water or wine lees powder (WLPW) and intraperitoneal injection of Saline or Sirtinol. Significant differences (*p* < 0.05) are represented by different letters and *p* was estimated by one-way ANOVA.

The effects of WL phenolic compounds on BP in rats treated with L-NAME, indomethacin, or sirtinol were investigated. Initial values of SBP and DBP in these animals were 193 ± 6.8 and 147 ± 8.2 mm Hg, respectively. Figure 2, Figure 3 and Figure 4 show the BP changes in SHR 6 h after oral administration of water or WLPW (125 mg/kg bw), and additionally treated intraperitoneally with saline solution, L-NAME, indomethacin, or sirtinol.

BP observed in the Water + Saline group did not change by the treatment. Nevertheless, as expected, the oral administration of WLPW (WLPW + Saline group) produced a significant decrease in both SPB and DBP (−28 ± 2.1 and −30 ± 8.02 mm Hg, respectively; *p* ≤ 0.05) with respect to Water + Saline group (Figure 2, Figure 3 and Figure 4).

When animals were intraperitoneally treated with L-NAME (30 mg/kg bw), it caused an increase in SBP of water group. No changes were observed in DBP. Regarding WLPW + L-NAME group, SBP and DBP values were similar to those observed for Water + Saline group, showing a total loss of WLPW antihypertensive effect observed in WLPW + Saline group (Figure 2).

No SBP and DBP changes were observed after intraperitoneal administration of indomethacin (5 mg/kg bw) and sirtinol (1 mg/kg bw) in the two respective water groups (Water + Indomethacin and Water + Sirtinol, respectively) (Figure 3 and Figure 4, respectively).

SBP values of WLPW + Indomethacin and WLPW + Sirtinol groups were significantly lower than the one showed by Water + Saline group, showing an antihypertensive effect. However, their BP-lowering effects were less potent to those observed by WLPW + Saline group (Figure 3A and Figure 4A, respectively). In the case of DBP, the values found in the WLPW + Indomethacin and WLPW + Sirtinol groups were similar to those found in their respective control groups (Water + Sirtinol and Water + Indomethacin, respectively) (Figure 3B and Figure 4B).

### 3.2. Endothelial-Related Gene Expression

**Figure 5:**
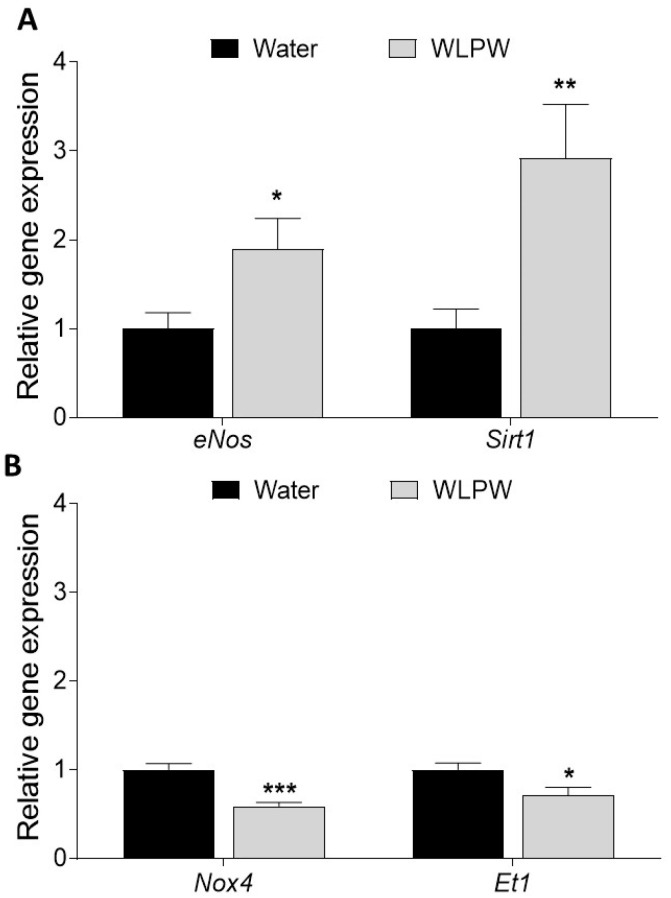
Aortic gene expression of *eNos* and *Sirt1* (**A**) and *Nox4* and *Et1* (**B**) in spontaneously hypertensive rats 6 h after administration with water or wine lees powder (WLPW). Statistical differences between treatments were carried out by Student’s *t*-test when (*) *p* < 0.05, (**) *p* < 0.01 or (***) *p* > 0.001.

In the second study, the expression of endothelial function-associated genes was studied at 6 h post-administration of water or WLPW to SHR (Figure 5). Administration of WLPW significantly upregulated the expression of *eNos* and *Sirt1* (1.9 and 2.9 times higher, respectively) (Figure 5A), while the expressions of *Et1* and *Nox4*, involved in a vasoconstrictor effect, were significantly downregulated by WLPW intake (Figure 5B).

## 4. Discussion

Dietary patterns based on the consumption of fruits and vegetables are associated with lower risk of HTN and an improvement of endothelial function [19]. These beneficial effects are mainly related to the phenolic compounds present in these foods [20,21,22]. In this sense, a previous study carried out by our group in SHR demonstrated that WLPW administration at an acute dose of 125 mg/kg bw to SHR produced a potent decrease on BP, showing the maximum effect at 6 h post-administration. This effectiveness was attributed to its high content in phenolic compounds, mainly flavanols and anthocyanins. In addition, it was evidenced that WLPW exerts its antihypertensive action via an improvement of oxidative stress since reduced levels of hepatic ROS and plasma malondialdehyde and increased levels of hepatic reduced glutathione (an endogenous antioxidant) were observed in the treated animals [15]. However, an improvement of endothelial function after WLPW administration should not be ruled out since it has been reported as one of the mechanisms involved in the antihypertensive effect of other winery by-products to hypertensive rats [17,23,24]. Therefore, the objective of this study was to elucidate the potential role of endothelial-derived factors and SIRT1 in the antihypertensive effect of WLPW. In order to achieve this goal, we treated SHR with L-NAME, an inhibitor of eNOS, indomethacin, an inhibitor of COX, and sirtinol, an inhibitor of sirtuin synthesis. Furthermore, the aortic expression levels of different genes involved in endothelial dysfunction were evaluated after WLPW administration in other additional experiment. Both studies were carried out at 6 h post-administration, since, at this time point, the maximum BP decrease caused by WLPW was observed [15].

Initially, we focused in studying the role of NO in the antihypertensive effect of WL phenolic compounds. NO is the main vasodilator factor produced and released by the endothelium via eNOS [4]. NO activates guanylyl cyclase that produces cyclic guanosine monophosphate. This molecule leads to relaxation of the muscle layer and vasodilation [4,25]. Due to this activity, animals were intraperitoneally administered L-NAME 4 h after oral administration of WLPW to inhibit the endothelial NO production. Its administration produced a total loss of the SBP- and DBP-lowering effects showed by WLPW. Therefore, these results provide clear evidence that antihypertensive effect of WLPW is NO-mediated in SHR. According to this, human clinical studies have shown that moderate consumption of aged white wine produces a higher bioavailability of NO, which was linked to a reduction in BP [26]. Similar to our results, these effects were attributed to the presence of grape-derived compounds in the aged white wine, such as polyphenols. In addition, our results are also in concordance with previous results published by our group with other grape phenolic-rich extracts and flavanol-rich food [24,27]. In this regard, Quiñones et al. and Pons et al. observed that the antihypertensive effect of an acute dose of 375 mg/kg bw of GSPE to SHR and to cafeteria diet-induced hypertensive rats, respectively, was totally abolished when animals were additionally treated with L-NAME [24,27]. The same effect was also observed in SHR treated with flavanol-rich cocoa in acute dose (300 mg/kg bw), when eNOS activity was inhibited [28].

Nevertheless, the BP-lowering effect of WLPW has been also associated with a high content of the phenol family of anthocyanins [14], which have also shown vascular benefits and their circulating metabolites have been directly related to these properties [29]. In this regard, the beneficial effect of anthocyanin rich foods or extracts on vascular health has been evidenced in a meta-analysis of randomised controlled trials [30]. Furthermore, the involvement of NO in the endothelium-dependent vascular relaxing effect of extracts rich in anthocyanins, and in flavanols, has also been corroborated ex vivo in vascular reactivity studies carried out in the presence of eNOS inhibitors [24,31].

Studies conducted with grape phenolic compounds using GSPE in a model of cafeteria diet-fed hypertensive rats and a red wine extract in SHR showed that these extracts increased aortic *eNos* expression [16,17,23]. Our findings are in line with these studies since the expression of aortic *eNos* of WLPW-treated SHR was higher than that shown by untreated animals, suggesting that WL phenolic compounds could increase NO availability, in turn increasing *eNos* expression. Although mRNA expression is not indicative of changes in protein levels, it is noteworthy that, in a previous study, we showed an increase of plasma NO levels after the administration of WLPW to SHR compared to SHR administered water [15]. In addition to eNOS levels, the increase of endothelial NO levels can be also due to changes in eNOS activity. SIRT1 promotes endothelium-dependent vasodilation by targeting eNOS for deacetylation, increasing the activity and/or expression of this enzyme [32]. It has been evidenced that SIRT1 is involved in the beneficial effects of some phenolic compounds present in grapes, wine, and grape by-products, such as proanthocyanidins, resveratrol, or quercetin [23,33]. Pons et al. reported that the antihypertensive effect of a grape seed proantocyanindin extract totally disappeared when SIRT1 activity was inhibited [23]. Specifically, the effect of the extract could be mediated by an upregulation of endothelial *Sirt1* expression since the aortic *Sirt1* expression was increased after its acute and chronic administration [17,23]. In our study, the antihypertensive effect of WLPW was partially abolished by SBP and totally by DBP, when animals were treated with WLPW and sirtinol. Furthermore, WLPW produced an upregulation of endothelial *Sirt1* (1.5 times higher than water group). These results showed that SIRT1 is clearly involved in the BP-lowering process produced by WLPW. Flavanols present in WLPW, similarly to GSPE, could be the bioactive compounds responsible of this effect on SIRT1. However, it should not be ruled out that other phenolic compounds could be involved. Kitada et al. observed an upregulation of peripheral blood mononuclear *SIRT1* expression in volunteers consuming a non-alcoholic red wine extract for eight weeks. The consumed daily dose contained 19.2 mg of resveratrol and 136 mg of other polyphenols including tanins, catechin, epicatechin, or quercetin glucoside [34]. In addition, resveratrol and quercetin, also present in WLPW, were also reported to be activators of SIRT1 in other studies [33].

In addition to the effect of SIRT1 on eNOS, this deacetylase also can modulate the activity and expression of some vascular NOX such as NOX4, one of the main producers of ROS in endothelium [8,35]. An overproduction of ROS linked to an oxidative stress state leads to reduce NO bioavailability since ROS scavenge NO to produce peroxynitrite [36], avoiding NO-induced vasodilation. There is evidence of the presence and possible involvement of oxidative stress in the antihypertensive process in SHR [37,38]. In a previous study of our group, the antioxidant properties of WLPW were demonstrated in SHR after its acute administration. WLPW caused a decrease in ROS levels and an increase in reduced glutathione (GSH) in liver as well as a decrease in MDA and increase of NO in plasma [15]. In order to know if an antioxidant effect would also be produced in the endothelium, we studied the levels of *Nox4* expression in aorta of SHR treated with WLPW at 6 h post-administration. Aortic *Nox4* expression was drastically reduced, suggesting an important implication of this endothelial enzyme in the effect of WL phenolic compounds. Since SIRT1 can downregulate *Nox4* [8] and WLPW intake produced an upregulation of *Sirt1* expression in the present study, the increase of expression of *Sirt1* could be one of the mechanisms involved in the increase of NO availability. Our findings are in agreement with the effect observed for other antihypertensive grape flavanols, which produced a downregulation of *Nox4* [17,23]. Furthermore, WLPW is rich in anthocyanins, which also may be involved in the downregulation of *Nox4*. Anthocyanins as malvidin, malvidin-6-glucoside or malvidin-6-galactoside from blueberry, also found in WLPW, have been demonstrated to reduce *Nox4* expression in high glucose-induced human umbilical cord vein endothelial cells (HUVECs) [39]. In addition, Galindo et al. have demonstrated that quercetin, also found in a great quantity in WLPW, reduces the aortic expression of *Nox4* after five weeks of administration [40].

Furthermore, it was studied the effect of WLPW in the production of endothelial ET-1. ET-1 is a potent vasoconstrictor and several studies have reported an overexpression of *Et1* in the endothelium in hypertensive state [41]. In this sense, our results showed a reduction of aortic *Et1* gene expression 6 h after administration of WLPW, rich in flavanols and anthocyanins (Figure 5), suggesting their implication in the reduction of BP. Food by-products extracts rich in flavanols such as GSPE have shown a reduction in aortic *Et1* mRNA expression levels in hypertensive rats [23,24]. Furthermore, the administration of food extracts rich in anthocyanins reduced endothelial expression of *ET1* in HUVECs, which were induced endothelial dysfunction by hyperglycemia [42]. Specifically, in the same cells without induced endothelial dysfunction, the anthocyanins delphinidin and cyanidin exerted a reduction of ET-1 secretion and expression, where the higher effect was demonstrated by delphinidin [43]. In addition, a relationship between ET-1 and NOX has been reported. In this regard, it has been observed that ET-1 increases the production of ROS in the vasculature through the activation of NOX [44,45]. A reduction in *Et1* and *Nox4* gene expression has been found after the administration of WLPW. All these findings showed that WL phenolic compounds could improve the endothelial dysfunction linked to HTN, balancing the endothelium-derived vasodilator and vasoconstrictor factors.

In addition to NO, PGI2 is another important endothelium-derived vasodilator involved in the regulation of BP, which is generated by the action of COX and prostacyclin synthase [11]. The administration of the COX inhibitor indomethacin to animals produces the inhibition of PGI2 production. Our results showed that the antihypertensive effect of WLPW is also mediated by PGI2 as animals treated WLPW and indomethacin showed a lower antihypertensive effect that the one observed in WLPW + Saline group. Similar results were found with GSPE. The antihypertensive effect of that extract was partially mediated by prostacyclin in SHR and cafeteria diet-induced hypertensive rats [24,27]. Moreover, SHR administered GSPE showed an increase of plasma PGF1 levels, which is a stable metabolite of PGI2 [24]. Nevertheless, results obtained in the same study from ex vivo experiments using aorta ring preparations showed that the vasodilator effect of GSPE in the aorta was not mediated by PGI2. These contradictory results between in vivo and in vitro experiments could be explained since the aorta is a conduit artery and resistance arteries determine the arterial blood pressure more than the large vessels [24]. Other studies have shown an increase in the release of PGI2 in procyanidin-treated human aortic endothelial cells [46]. In addition, increased levels of prostacyclin have been observed in the plasma of rats [47,48] and humans [46] after the consumption of flavanols.

## 5. Conclusions

**Figure 6:**
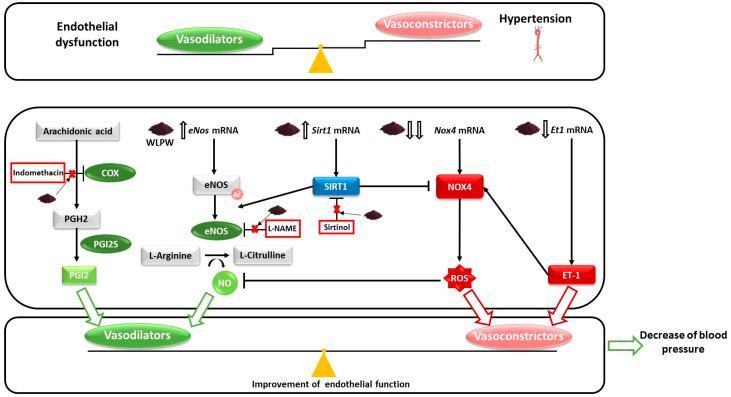
Schematic representation of the molecular mechanisms involved in the blood pressure (BP)-lowering effect of wine lees powder (WLPW) in endothelium. WLPW upregulates endothelial nitric oxide (*eNos*) mRNA levels, which would lead to higher production of nitric oxide (NO). WLPW increases plasma NO levels [15]. Sirtuin-1 (*Sirt1*) mRNA levels are also upregulated by WLPW. The enzyme SIRT1, which mediates partially the antihypertensive effect of WLPW, demonstrated after sirtinol administration, is able to increase *eNos* expression. In addition, SIRT1 deacetylates and activates eNOS and inhibits NADPH oxidase subunit 4 (NOX4). Furthermore, *Nox4* mRNA levels were downregulated by WLPW. The decrease in the expression and activity of NOX4 would lead to a decrease in radical oxygen species (ROS) production and consequently, an increase in NO availability. All these events would lead to the increase of NO availability, which mediates the antihypertensive effect of WLPW, as it has been demonstrated after L-NAME administration. WLPW exerts an improvement of oxidative stress since reduces levels of hepatic ROS and plasma malondialdehyde and increases levels of hepatic reduced glutathione [15]. In addition, the indomethacin study has demonstrated that the endothelium-derived vasodilator factor prostaglandin I2 (PGI2) mediates partially the BP-lowering effect of WLPW. Moreover, WLPW also downregulates the expression of the vasoconstrictor endothelin-1 (*Et1*) mRNA levels, which would lead to lower production of NO. All of these factors lead to a restoration of the imbalance of endothelial-derived vasodilator and vasoconstrictor factors caused by hypertension, improving endothelial function and decreasing BP. Arrows ending in points represent activation and those ending in lines represent inhibition. Red crosses indicate that WLPW antihypertensive effect disappears totally or partially when animals are further treated indomethacin, L-NAME or sirtinol.

In this study, we demonstrated the implication of NO and SIRT1 in the antihypertensive effect exerted by WLPW in SHR (Figure 6). WL phenolic compounds increase *eNos* and *Sirt1* mRNA levels in the endothelium and could tend to increase the eNOS activity. Furthermore, WL phenolic compounds also reduce the endothelial expression of *Nox4* and *Et1*, which would reduce endothelial ROS production and therefore could tend to increase the availability of vascular NO as well as the vasoconstrictor ET-1. In addition, a partial involvement of PGI2 in the antihypertensive effect of WLPW is also found, although more studies are needed to understand this effect in greater depth. Thus, WL phenolic compounds improve endothelium functionality and reduce BP in SHR. All these results suggest that WLPW could be a potential functional food ingredient with many benefits in the treatment of cardiovascular disease.

## 6. Patents

Patent application “Wine lees, derivatives thereof and their uses”: application number EP20382358.8 and PCT/EP2021/053051.

## Figures and Tables

**Table 1:** Primer list characteristics.

Rat Primers	Sequence (5′…3′)	Amplicon Size	Efficiency	GenBank Accesion No.
*eNOS Fw*	GGATTCTGGCAAGACCGATTAC	159	2.23 (111.5%)	NM_021838.2
*eNOS Rv*	GGTGAGGACTTGTCCAAACACT			
*Sirt1 Fw*	TTGGCACCGATCCTCGAA	217	1.97 (98.5)	XM_006223877.1
*Sirt1 Rv*	ACAGAAACCCCAGCTCCA			
*Nox4 Fw*	GTGTCTGCATGGTGGTGGTA	150	1.86 (93%)	NM_053524.1
*Nox4 Rv*	TCAACAAGCCACCCGAAACA			
*Et1 Fw*	TGATTCTCTTGCCTCTTCTTG	110	2.23 (111.5%)	NM_012548.2
*Et1 Rv*	TATGGAATCTCCTGGCTCTC			
*Ppia Fw*	CTTCGAGCTGTTTGCAGACAA	118	2.28 (114%)	NM_017101.1
***Ppia Rv***	AAGTCACCACCCTGGCACATG			

## Data Availability

Data is contained within the article and supplementary material.

## Supplementary Materials

The following are available online at https://www.mdpi.com/article/10.3390/antiox10071073/s1, Table S1: Non-anthocyanin composition of wine lees powder obtained by UHPLC-(ESI-)-Q-TOF-MS, Table S2: Anthocyanin composition of wine lees powder obtained by UHPLC-(ESI-)-Q-TOF-MS.
