# Supplementary material for: Blood Pressure-Lowering Effect of Wine Lees Phenolic Compounds Is Mediated by Endothelial-Derived Factors: Role of Sirtuin 1
Source: Antioxidants (Basel). 2021 Jul 3;10(7):1073. doi: 10.3390/antiox10071073 (PMC8301028; doi:10.3390/antiox10071073)
Supplement: Supplementary file 1 [file antioxidants-10-01073-s001.zip › antioxidants-1272829-supplementary.pdf]

Table S1. Non-anthocyanin composition of wine lees powder obtained by UHPLC-(ESI-)-Q-TOF-MS

Compound	Quantity (μg/g)
**Flavanols**	
Catechin	3,599.26 ± 17.70
Catechin gallate	29.49 ± 0.37
Epicatechin	1,602.95 ± 5.64
(Epi)catechin *O*-glucoside iso1	18.43 ± 0.01
(Epi)catechin *O*-glucoside iso2	12.17 ± 0.00
(Epi)catechin *O*-glucoside iso3	54.19 ± 1.08
Procyanidin dimer B2	1,275.58 ± 0.37
Procyanidin dimer iso1	2,367.19 ± 5.64
Procyanidin dimer iso2	525.71 ± 2.30
Procyanidin dimer iso3	109.12 ± 0.76
Procyanidin dimer iso4	471.89 ± 3.92
Procyanidin dimer iso5	159.26 ± 0.82
Procyanidin trimer iso1	600.18 ± 3.80
Procyanidin trimer iso2	529.03 ± 9.77
Procyanidin trimer iso3	225.25 ± 2.14
Procyanidin trimer iso4	118.71 ± 3.90
Procyanidin trimer iso5	508.39 ± 3.51
**Flavonols**	
Quercetin	1,355.94 ± 4.47
Quercetin-3-*O*-glucoside	60.09 ± 0.39
Quercetin-3-*O*-glucuronide	89.22 ± 0.76
Kaempferol	189.86 ± 1.50
Kaempferol-3-*O*-glucuronide	17.70 ± 0.35
Isorhamnetin	411.43 ± 2.31
**Phenolic acids**	
Gallic acid	4,456.04 ± 89.03
Caffeic acid	120.55 ± 0.77
Caffeic acid *O*-glucoside iso1	20.28 ± 0.74
Caffeic acid *O*-glucoside iso2	24.33 ± 1.11
p-Coumaric acid	126.82 ± 0.68
4-Hydroxybenzoic acid	61.57 ± 2.14
Ferulic acid	27.65 ± 0.43
Vanillic acid	85.90 ± 2.41
**Stilbenes**	
trans-Resveratrol	169.59 ± 0.74
Resveratrol iso1	108.76 ± 0.37
Resveratrol *O*-glucoside iso1	9.95 ± 0.37
Resveratrol *O*-glucoside iso2	49.77 ± 1.47
Piceatannol	154.84 ± 2.00
Piceatannol 3-*O*-glucoside iso1	8.11 ± 0.00
Piceatannol 3-*O*-glucoside iso2	2.21 ± 0.00
Viniferin-iso1	9.95 ± 0.00
Viniferin-iso2	29.86 ± 0.41

Data are represented per g of wet weight.

Table S2. Anthocyanin composition of wine lees powder obtained by UHPLC-(ESI-)-Q-TOF-MS

Anthocyanins	Quantity (μg/g)
Gallocatechin-malvidin-3-glucoside dimer	9.09 ± 0.04
Malvidin-3-glucoside-(epi)catechin	40.95 ± 0.12
Delphinidin-3-glucoside	136.02 ± 1.68
Cyanidin-3-glucoside	8.34 ± 0.75
Petunidin-3-glucoside	185.45 ± 2.08
Petunidin-3-glucoside-pyruvic acid	3.28 ± 0.04
Peonidin-3-glucoside	100.31 ± 2.30
Malvidin-3-glucoside	2,236.82 ± 18.44
Peonidin-3-glucoside-pyruvic acid	1.50 ± 0.03
Delphinidin-(6-acetyl)-3-glucoside	33.48 ± 0.83
Visitin A (malvidin-3-glucoside-pyruvic acid)	45.23 ± 0.12
Visitin B (malvidin-3-glucoside-acetaldehyde)	112.92 ± 0.54
Malvidin-3-glucoside-ethyl-(epi)catechin	13.47 ± 0.03
Cyanidin-(6-acetyl)-3-glucoside	7.50 ± 0.21
Acetylvisitin A	28.95 ± 0.41
Malvidin-3-glucoside-ethyl-(epi)catechin	50.75 ± 0.22
Petunidin-(6-acetyl)-3-glucoside	47.51 ± 1.72
Malvidin-3-glucoside-ethyl-(epi)catechin	75.36 ± 0.67
Acetylvisitin B	61.24 ± 0.41
Peonidin-(6-acetyl)-3-glucoside	48.65 ± 1.14
Delphinidin-(6-coumaroyl)-3-glucoside	16.10 ± 0.25
Malvidin-(6-acetyl)-3-glucoside	1,046.67 ± 0.77
Coumaroylvisitin A	7.38 ± 0.06
Malvidin-(6-caffeoyl)-3-glucoside	13.42 ± 0.25
Cyanidin-(6-coumaroyl)-3-glucoside	3.65 ± 0.15
Catechin-ethyl-Malvidin-3-acetylglucoside dimer	32.34 ± 0.29
Petunidin-(6-coumaroyl)-3-glucoside	27.46 ± 0.33
Pinotin A (malvidin-3-glucoside-vinylcatechol)	30.96 ± 0.47
Malvidin-glucoside-vinyl-catechin	5.64 ± 0.03
Coumaroylvisitin B	33.62 ± 0.26
Malvidin-3-glucoside-vinylguaiacol	21.92 ± 0.18
Catechin-ethyl-malvidin-3-coumaroylglucoside dimer	25.24 ± 0.10
Catechin-ethyl-malvidin-3-acetylglucoside dimer	5.33 ± 0.06
Peonidin-(6-coumaroyl)-3-glucoside	34.82 ± 1.00
Malvidin-(6-coumaroyl)-3-glucoside	396.97 ± 0.55
Malvidin-glucoside-vinyl-catechin	5.99 ± 0.02
Acetyl-pinotin A	0.26 ± 0.00
Malvidin 3-*O*-glucoside 4-vinylphenol (Pigment A)	23.58 ± 0.07
Catechin-ethyl-malvidin-3-coumaroylglucoside dimer	4.37 ± 0.01
Malvidin acetyl 3-*O*-glucoside 4-vinylphenol (Acetyl-pigment A)	14.12 ± 0.16

Data are represented per g of wet weight.
